# Oncologist-led *BRCA* ‘mainstreaming’ in the ovarian cancer clinic: A study of 255 patients and its impact on their management

**DOI:** 10.1038/s41598-020-60149-5

**Published:** 2020-02-25

**Authors:** Megan Rumford, Mark Lythgoe, Iain McNeish, Hani Gabra, Laura Tookman, Nazneen Rahman, Angela George, Jonathan Krell

**Affiliations:** 1 National Heart and Lung Institute, Faculty of Medicine, Imperial College, London, UK; 2 Department of Medical Oncology, Hammersmith Hospital, London, UK; 3 Department of Surgery and Cancer, Imperial College, London, UK; 4 Cancer Genetics Unit, Royal Marsden National Health Service Foundation Trust, London, United Kingdom; 5 Division of Genetics and Epidemiology, Institute of Cancer Research, London, United Kingdom; 6 Gynaecology Unit, The Royal Marsden NHS Foundation Trust, London, UK

**Keywords:** Molecular medicine, Ovarian cancer

## Abstract

Although guidelines recommend BRCA testing for all women with non-mucinous epithelial ovarian cancer, there is significant variability in access to testing across the UK. A germline B*RCA* mutation (*BRCA*m) in ovarian cancer patients provides prognostic and predictive information and influences clinical management, such as the use of PARP inhibitors, which have demonstrated a progression-free survival benefit in the *BRCA*m cohort. Additionally, the finding of a *BRCA*m has significant implications for patients and their families in terms of cancer risk and prevention. We studied the impact of a newly-formed, oncologist-led ‘mainstreaming’ germline *BRCA* testing pathway in 255 ovarian cancer patients at Imperial College NHS Trust. Prior to the establishment of ‘mainstreaming’, uptake of germline *BRCA* testing was 14% with a mean turnaround time of 148.2 calendar days. The ‘mainstreaming’ approach led to a 95% uptake of germline *BRCA* testing and a mean turnaround time of 20.6 days. Thirty-four (13.33%) *BRCA*m patients were identified. At the time of data collection nine *BRCA*m patients had received a PARP inhibitor off-trial, three had entered a PARP inhibitor trial and 5 were receiving platinum-based chemotherapy with a plan to receive PARP inhibitor maintenance. This study provides further evidence of the impact of oncologist-led ‘mainstreaming’ programs.

## Introduction

In the United Kingdom (UK), over 7,000 cases of ovarian cancer are diagnosed annually, resulting in over 4,000 deaths per year – making ovarian cancer the 6^th^ most common cancer in women^1^. There is a well-documented association between women carrying pathogenic germline mutations in breast cancer gene one (*BRCA1*) or breast cancer gene two (*BRCA2*) and their risk of developing ovarian cancer^2,3^. Identifying ovarian cancer patients carrying germline breast cancer gene (*BRCA*) mutations provides important insight into why the patient may have developed ovarian cancer, is indicative of the patient’s prognosis and can help determine the optimal therapeutic interventions and best approach to clinical management of their disease^4,5^.

### Characteristics of *BRCA*-mutated (*BRCA*m) ovarian cancer and impact of *BRCA* status on clinical decision making

Previous studies indicate that the prevalence of germline *BRCA* mutation carriers in a non-mucinous ovarian cancer cohort is around 13–15%^5–9^. The mean age of diagnosis of ovarian cancer in patients carrying a *BRCA1* mutation can be five to ten years lower than the mean age of diagnosis of *BRCA* wild-type (*BRCA*wt) patients (patients carrying *BRCA2* mutations do not show such a marked difference in age of diagnosis from the *BRCA*wt population)^7,9,10^. Literature also suggests that *BRCA*m ovarian cancer is more likely to be diagnosed at an advanced stage of disease^6^, where survival outcomes are poorer.

There are significant differences in the response to treatment and survival outcomes between *BRCA*m and *BRCA*wt ovarian cancer populations, even when the same therapeutic intervention is applied irrespective of *BRCA* status^6,11^, resulting in greater median progression-free survival and median overall survival times in the *BRCA*m patient cohort^6,12^. Additionally, a patient’s *BRCA* status can inform decision making around risk-reducing procedures for both the individual and any subsequently-identified *BRCA* mutation carriers within the family^5,11–13^. Identifying further *BRCA*m carrier family members via cascade testing has also been demonstrated to be a cost-effective mechanism to prevent future cancers in relatives^5,14^.

Although the presence of a *BRCA* mutation is considered a predictive biomarker for response to treatment^13^, prior to the introduction of poly ADP-ribose polymerase (PARP) inhibitors into clinical practice the knowledge of a patient’s *BRCA* status had little impact on the management of their ovarian cancer. In December 2014 PARP inhibitor olaparib received European Medicines Agency (EMA) Marketing Authorisation for the treatment of relapsed, platinum-sensitive *BRCA*m ovarian cancer^15^ and was made available by the National Health Service (NHS) in early 2016^16^. The availability of this targeted therapy for *BRCA*-mutated patients created a shift towards a more personalised approach to the management of ovarian cancer and further highlighted the need for *BRCA* testing^5,6,13,17,18^. Following the approval of olaparib, clinical utility has also been demonstrated in both *BRCA*m and *BRCA*wt cohorts with other licenced PARP inhibitors, niraparib and rucaparib^19,20^.

The announcement of the SOLO1 trial results^21^ in October 2018 has impacted further on the approach to treating *BRCA*m ovarian cancer. Results of this study show that patients receiving olaparib maintenance in the first line setting benefit from an estimated three year median progression free survival advantage versus those *BRCA*m patients who received placebo^21^. The recent licence extension for olaparib to allow maintenance treatment in the first line setting further highlights how knowledge of a patients *BRCA* status at the outset of their disease management has become increasingly important.

### Access to *BRCA* testing in the United Kingdom

There is an overwhelming body of literature advocating that germline *BRCA* testing should be offered to all women with a diagnosis of non-mucinous ovarian cancer, irrespective of age at diagnosis or family history of *BRCA*-associated cancer^5–7,9,17,22^. The NICE Clinical Guideline on Familial Breast Cancer (CG164) recommends that *BRCA* testing be offered to any woman with ovarian cancer where the probability of carrying a *BRCA* mutation is 10% or greater^23^ – considering that the prevalence of *BRCA* mutation carriers in non-mucinous ovarian cancer cohorts is around 13–15%^5–9^, this would indicate that all patients with a diagnosis of non-mucinous ovarian cancer should be offered *BRCA* testing. This is further supported by the recommendation of the Independent Cancer Taskforce, who suggest that *BRCA* testing should be offered *at the point of diagnosis* to the aforementioned patient group^24^. Despite a clear clinical and scientific rationale, the implementation of these guidelines is at the discretion of each regional genetics centre – which has resulted in significant variability of access to *BRCA* testing across the UK^13^. Furthermore, there are currently no guidelines recommending somatic *BRCA* testing for women with ovarian cancer and currently there is no NHS funding for this in the UK.

Although relatively new, there is a growing body of evidence supporting the feasibility of an oncologist-led ‘mainstreaming’ approach^5,8,17,18^ – where the oncology team introduce the concept of genetic testing and obtain patient consent as part of routine appointments, deliver initial results and then refer any identified *BRCA*m patients or those with a Variant of Unknown Significance (VUS) on to specialised genetic departments for counselling and cascade testing of potentially affected family members.

### Purpose of this study

With the increased clinical utility of *BRCA* testing and broader availability of PARP inhibitors for *BRCA*m patients, the landscape of ovarian cancer treatment is changing. This study aims to assess the impact of the introduction of an oncologist-led *BRCA* testing mainstreaming service at Imperial College Healthcare NHS Trust on 255 ovarian cancer patients, previously untested for germline *BRCA* mutations and to examine the epidemiology of the *BRCA*m and *BRCA*wt cohorts within this population, evaluate the impact of the knowledge of the patients’ *BRCA* status on clinical decision-making, and provide further evidence of the utility and success of this pathway.

In light of conflicting opinions on the implementation of an age-based threshold for access to *BRCA* testing, an additional aim of this study is to investigate the prevalence of *BRCA*m in older patients (diagnosis ≥ 70 years), and any subsequent impact a *BRCA* mutation status has on their clinical management.

## Methods

### Patient selection, consent to *BRCA* testing and ongoing clinical management

All patients with a confirmed diagnosis of non-mucinous ovarian cancer and an unknown *BRCA* status who were under the care of the gynaecological oncology team at Imperial College Healthcare NHS Trust, on or after the 1^st^ April 2016, were eligible for germline *BRCA* testing through the Imperial College Hospital Mainstreaming Programme (ICHMP). During the time of this study we identified 312 patients at ICHNT with non-mucinous ovarian cancer. Of this cohort, 14% had already undergone *BRCA* testing via alterative mechanisms. Of the 268 untested patients who remained eligible for testing, there was a 95% uptake of BRCA testing via the mainstreaming route. The remaining 5% either declined *BRCA* testing or were deceased prior to *BRCA* testing taking place.

The ICHMP could be introduced, and the patient subsequently consented for germline *BRCA* testing, by any member of the gynaecological oncology team who had completed the Mainstreaming Cancer Genetics online training program (available at http://www.mcgprogramme.com/BRCAtoolkit/). This could occur at any scheduled appointment during the patient’s treatment or routine surveillance. The consenting process involved discussion of what the *BRCA* gene is, what a mutation and VUS is and what the relevance of the finding of a mutation might be to the patient (in terms of treatment of their ovarian cancer and future screening and prevention for other *BRCA*-associated disease) as well as the relevance of the finding to other blood relatives. Patients were given time to decide on whether they wished to proceed with *BRCA* testing. Patients were also provided with written information developed by the oncology and genetics teams at Hammersmith Hospital and the Royal Marsden Hospital.

The first 32 patients tested via the ICHMP were consented following a group consenting process. They had responded to a written invitation from the oncology team to attend a lecture on *BRCA* testing and were subsequently offered a consultation and blood test that day if they agreed to it. This method was used to study whether such an approach could reduce the number of untested patients in the prevalent pool of patients within the ovarian cancer clinic.

Patients who received a *BRCA*m or VUS result were referred on to The Royal Marsden genetics department for genetic counselling and follow up. Patients with a negative result but a significant family history of cancer were also offered an appointment with a genetic counsellor if they wished. Clinical management of the patient’s disease continued under the gynaecological oncology team at Imperial College Healthcare NHS Trust.

All patients who underwent testing via the ICHMP between April 2016 and April 2018 (inclusive) were included in this analysis.

### *BRCA* testing methodology and recording of results

All germline *BRCA* testing was undertaken by TGLclinical (Institute of Cancer Research, 15 Cotswold Road, Sutton SM2 5NG). Samples were tested using the *Illumina TruSight Cancer Panel*. Although panel gene testing was undertaken, no pathogenic mutations or rare variants other than in *BRCA1* and *BRCA2* were released or reported, as was explained to patients prior to consenting to *BRCA* testing. Further information on the methodologies, analysis and reporting procedures can be found at www.TGLclinical.com.

### Collection of patient data

All patient data was obtained via a review of patient clinical records in the Imperial College Healthcare NHS Trust record management systems. Patient data has been collected as it was recorded by the treating clinicians.

## Results

### Clinical characteristics of the patient cohort

Patients who underwent testing via the ICHMP ranged from those who were newly-diagnosed to those who had undergone several relapses and multiple lines of prior therapy. Of the 255 patients tested, 34 (13.33%) patients were found to be carrying a germline *BRCA* mutation - 19 *BRCA1* mutation carriers and 15 *BRCA2* mutation carriers were identified (Table 1). No patients with a VUS were identified.

**Table 1:** Identified *BRCA* mutations.

Gene	Identified mutation	Number of affected patients
*BRCA1*	c.1066 C > T	1
*BRCA1*	c.174_1755del7	1
*BRCA1*	c.1898delC	1
*BRCA1*	c.1961dupA	2
*BRCA1*	c.315 T > A	1
*BRCA1*	c.4035delA	1
*BRCA1*	c.4399delC	1
*BRCA1*	c.4484 + 1 G > A	1
*BRCA1*	c.4484 G > A	1
*BRCA1*	c.4508 C > A	1
*BRCA1*	c.5080 G > T	1
*BRCA1*	c.5266dupC	1*
*BRCA1*	c.68_69delAG	4*
*BRCA1*	Exon 13 duplication	1
*BRCA1*	Exon 3–24 deletion	1
*BRCA2*	c.3599_3600delGT	1
*BRCA2*	c.4631delA	1
*BRCA2*	c.5054 C > A	1
*BRCA2*	c.5576.5579delTTAA	2
*BRCA2*	c.5946delT	1*
*BRCA2*	c.6486_6489delACAA	1
*BRCA2*	c.658_659delGT	1
*BRCA2*	c.7679_7680delTT	1
*BRCA2*	c.7761_7785del25	1
*BRCA2*	c.7980 T > G	1
*BRCA2*	c.8331 + 2 T > C	1
*BRCA2*	c.8575delC	1
*BRCA2*	c.9435_9436delGT	1
*BRCA2*	c.9682delA	1

^*^Denotes Ashkenazi Jewish founder mutations (35).

The mean age of diagnosis was 57.8 years for the *BRCA*m cohort and 62.9 years for the *BRCA*wt cohort (Table 2). The mean age of diagnosis for *BRCA1* carriers was 54.2 years, whilst the mean age of diagnosis for *BRCA2* carriers was 62.3 years. Fifteen (44.1%) *BRCA*m patients were aged ≥60 years at the time of diagnosis, with five (14.7%) *BRCA*m patients aged ≥70 years at the time of diagnosis. The mean time between diagnosis and obtaining a *BRCA* test result was 28.36 months (range 0–312 months).This was as due to the fact that although many patients in this study underwent *BRCA* mainstreaming at diagnosis, some patients within the study underwent *BRCA* testing at relapse having not had *BRCA* testing prior to the implementation of the ICHMP.

**Table 2:** Summary of patient clinical characteristics.

Patient/disease characteristic	Whole cohort (n = 255)	*BRCA*m (n = 34)	*BRCA*wt (n = 221)
Mean age at diagnosis* (range)	62.2 (31–91)	57.8 (39–78)	62.9 (31–91)
Mean age at time of *BRCA* testing (range)	64.6 (34–91)	60.4 (40–79)	65.2 (34–91)
**Histology, n (%)**			
High Grade Serous	197 (77.3%)	34 (100%)	163 (73.8%)
Carcinosarcoma	8 (3.1%)	0 (0%)	8 (3.6%)
Clear cell	14 (5.5%)	0 (0%)	14 (6.3%)
Endometroid	25 (9.8%)	0 (0%)	25 (11.3%)
Mixed	7 (2.7%)	0 (0%)	7 (3.2%)
Other or not available	4 (1.6%)	0 (0%)	4 (1.8%)
**Stage**^**#**^**, n (%)**			
I	53 (20.8%)	1 (2.9%)	52 (23.5%)
II	28 (11.0%)	1 (2.9%)	27 (12.2%)
III	93 (36.5%)	24 (70.6%)	69 (31.2%)
IV	76 (29.8%)	8 (23.5%)	68 (30.8%)
Not available	5 (2.0%)	0 (0%)	5 (2.3%)
**Lines of prior therapy at time of** ***BRCA*** **testing^, n (%)**			
N/A - about to commence adjuvant chemotherapy	46 (18.0%)	6 (17.6%)	40 (18.1%)
1	154 (60.4%)	17 (50.0%)	137 (62.0%)
2	32 (12.5%)	5 (14.7%)	27 (12.2%)
3	10 (3.9%)	4 (11.8%)	6 (2.7%)
4+	13 (5.1%)	2 (5.9%)	11 (5.0%)

^*^Diagnosis was based on histological confirmation of ovarian cancer, where the date of diagnosis is reflective of the date the histological sample was taken. Where no histological sample was taken, or records unavailable, the date of diagnosis reflects the date where the multidisciplinary team discussed the case and agreed to treat as ovarian cancer.

^Previous lines of therapy include all modalities of systemic or surgical treatment, where the ‘line’ of therapy had been either commenced or completed at the time of testing. Patients who had undergone primary surgery but had not undergone adjuvant chemotherapy were recorded as N/A.

^#^The stage of diagnosis was recorded using International Federation of Gynaecology and Obstetrics criteria.

### Clinical characteristics of the *BRCA*m cohort

Of the 34 identified *BRCA*m patients, five (14.7%) had prior breast cancer, one (2.9%) had prior oesophageal cancer, 26 (76.5%) had no prior cancer, and two (5.9%) patients had an unknown/undocumented personal cancer history. When considering these patients’ family history of *BRCA*-associated cancer (breast, gynaecological, pancreas or prostate), 15 (44.1%) reported a family history of *BRCA*-associated cancer, 13 (38.2%) reported no family history and six (17.6%) had an unknown/undocumented family history. Three patients (8.8%) had both a personal and family history of *BRCA*-associated cancer.

Twenty-nine different *BRCA* mutations were identified amongst the 34 patients, with three mutations identified in more than one patient. Six founder mutations of Ashkenazi Jewish origin were identified within the cohort.

### Impact of *BRCA*m status on patient management

At the time of data collection nine of the 34 identified *BRCA*m patients had received a PARP inhibitor as a direct consequence of the identification of a *BRCA* mutation. Six of these nine patients received olaparib post third-line therapy through NHS reimbursement, two received niraparib and one received rucaparib after second-line therapy via Early Access Programs. A further five *BRCA*m patients were receiving platinum-based chemotherapy for relapsed disease, where the intent of the clinical team was to initiate treatment with a PARP inhibitor following the completion of chemotherapy, assuming that a response to therapy was achieved. Fifteen *BRCA*m patients were either still receiving first-line (adjuvant) treatment or were in remission, and therefore not eligible to receive a PARP inhibitor at that time. Three of these patients receiving first-line therapy were subsequently recruited to the PRIMA trial (NCT02655016), a study of niraparib maintenance treatment in patients with advanced ovarian cancer following response on first-line platinum-based chemotherapy. The finding of a *BRCA* mutation influenced their decision to take part in this trial given the potential access to a PARP inhibitor maintenance therapy in the first-line setting. Five *BRCA*m patients were ineligible to receive PARP inhibitors either due to poor performance status (n = 2), platinum resistant disease at a time when there was no access to PARP inhibitors in this setting (n = 2) or death prior to further therapy (n = 1). Summary information on the characteristics of the *BRCA*m patients who received a PARP inhibitor can be seen in Table 3.

**Table 3:** Summary of disease characteristics and PARP inhibitor treatment characteristics for *BRCA*m patients in receipt of a PARP inhibitor (n = 9).

Disease stage	PARP inhibitor received	Number of cycles dispensed	Days on PARP inhibitor	Reason for discontinuation	Age at date of initiation on PARP inhibitor
Post third-line chemotherapy, platinum sensitive	Olaparib	8	210	Disease progression	59
Post third-line chemotherapy, platinum sensitive	Olaparib	7*	206	Disease progression	52
Post second-line chemotherapy, platinum sensitive^	Niraparib	4*	181	Disease progression	66
Post third-line chemotherapy, platinum sensitive	Olaparib	1	1	Disease progression	57
Post third-line chemotherapy, platinum sensitive	Olaparib	2	35	Disease progression	63
Post third-line chemotherapy, platinum sensitive	Olaparib	1 (ongoing)	Ongoing	N/A - ongoing	47
Post second-line chemotherapy, platinum sensitive^	Niraparib	3*	147	Disease progression	52
Post second-line chemotherapy, platinum resistant^	Rucaparib	2* (ongoing)	Ongoing	N/A - ongoing	77
Post third-line chemotherapy, platinum sensitive	Olaparib	1*	<7	Unacceptable toxicities	80

^patients accessed PARP inhibitor through Early Access Programs and/or Compassionate Use Programs initiated by sponsoring company (all other patients accessed PARP inhibitor via the NHS, as per NICE guidelines).

*patients received dose interruption and/or modification in order to manage adverse events/toxicities.

Of the 221 *BRCA*wt patients who underwent germline *BRCA* testing via the ICHMP, five received niraparib. This was via the Early Access Program which provided niraparib as a maintenance therapy for patients with platinum-sensitive relapsed ovarian cancer who were in response to further platinum therapy.

### Uptake and utility of mainstreaming pathway

There was a 95% uptake of *BRCA* testing via the ICHMP. The remaining 5% of patients either declined *BRCA* testing or were deceased prior to *BRCA* testing taking place. This compared to a figure of 14% of patients who underwent *BRCA* testing prior to the implementation of the ICHMP, when patients required referral to the regional genetics centres for counselling and consideration of *BRCA* testing.

Turnaround time was assessed in the 199 samples where both the date of sample acquisition and the date of test result was recorded. The mean turnaround time between blood sample acquisition and return of *BRCA* result to the treating oncologist was 20.6 calendar days, with a range of 11–42 calendar days. This compares favourably to the mean turnaround time of 148.2 calendar days prior to the implementation of the ICHMP.

## Discussion

### Epidemiology of Imperial College Healthcare NHS Trust cohort, and comparisons to other published literature

When considering the epidemiology of this cohort, these results are generally in line with other cohorts of ovarian cancer patients who have undergone systematic *BRCA* testing at other centres in the UK or abroad^5–8,11^. The age of diagnosis is lower in the *BRCA*m population than *BRCA*wt population, which reflects one of the hallmarks of *BRCA*-associated disease^6,7,9^. All *BRCA*m patients in this cohort had high grade serous histology. Although it is unusual that no other histologies were detected in the *BRCA*m population in this cohort, it is to be expected that the majority would have high grade serous histology given that this was the most common histology observed in other non-mucinous ovarian cancer cohorts^5,11^. The absence of any other histological subtypes is likely attributable to the relatively small sample size of this population. We would still advocate *BRCA* testing in all non-mucinous subtypes given the observation of *BRCA* mutations in non-mucinous, non-high grade serous groups in other studies^5,11^. These results are also consistent with previous findings that *BRCA*m ovarian cancer is more likely to be diagnosed at an advanced stage of disease^6^. Comparisons to other similarly-sized UK cohorts can be seen in Tables 4 and 5.

**Table 4:** Comparison of results from this study versus published results from other similar studies undertaken in other sites in the South of England.

Study result	Site of study			
	ICHMP cohort	The Royal Marsden (George *et al*.^5^)	University College London Hospital (Rahman *et al*.^8^)	East Anglia (Plaskocinska *et al*.^11^)
Number of patients	255	207	122	232
Criteria for testing	Epithelial ovarian cancer	Non-mucinous ovarian cancer	High grade non-mucinous ovarian cancer	Newly-diagnosed epithelial ovarian cancer
*BRCA*m, % (n)				
	13.33% (34)	15.94% (33)	14.75% (18)	7.76% (18)
VUS, % (n)	0% (0)	0% (0)	7.38% (9)	6.47% (15)
Age of diagnosis *BRCA*wt, years (range)	Mean 62.9 (31–91)	Mean 57.8 (22–81)	Median 62 (28–88)	Mean 66.1 (28–89)
Age of diagnosis *BRCA*m, years (range)	Mean 57.8 (39–78)	Mean 53.9 (30–87)	Median 58 (42–74)	Mean 49.5 (40–75)
Turnaround time of testing service	Mean 20.6 calendar days (range 11–42)	Not stated	Median 26 working days (range 14–48)	Median 46 calendar days (range 15–177)

**Table 5:** Comparison of *BRCA*m population results from this study versus reported results from similar studies undertaken in other sites in the South of England.

Study characteristic, *BRCA*m population	Site of study			
	Imperial College Healthcare NHS Trust	The Royal Marsden (George *et al*.^5^)	University College London Hospital (Rahman *et al*.^8^)	East Anglia (Plaskocinska *et al*.^11^)
Number of *BRCA*m patients	34	33	18	18
Personal breast cancer history, n (%)	6 (17.6%)	2 (6.1%)	Not stated	6 (33.3%)
Family history of breast, ovarian or other relevant cancer, n (%)	15/28* (53.6%)	16 (48.5%)	9/13* (69.2%)	Not stated
**Histology**				
Serous	34 (100%)	32 (97.0%)	17 (94.4%)	15 (83.3%)
Carcinosarcoma	0 (0%)	0 (0%)	1 (5.6%)	0 (0%)
Adenocarcinoma	0 (0%)	0 (0%)	0 (0%)	2 (11.1%)
Endometroid	0 (0%)	1 (3.0%)	0 (0%)	1 (5.6%)
**Stage**				
I	1 (2.9%)	0 (0%)	0 (0%)	4 (22.2%)
II	1 (2.9%)	4 (12.1%)	1 (5.6%)	0 (0%)
III	24 (70.6%)	25 (75.8%)	10 (55.6%)	12 (66.7%)
IV	8 (23.5%)	4 (12.1%)	7 (38.9%)	2 (11.1%)
Not available	0 (0%)	0 (0%)	0 (0%)	0 (0%)

^*^Remaining patients in cohort did not have any information on family history recorded, so were omitted from this analysis.

This study reports that approximately half of the *BRCA*m carriers do not have any relevant family history of *BRCA*-associated cancer (or at least, are not aware or cannot recall any family history in order to communicate this to their care team), which is similar to other cohorts in the UK (Table 5) and abroad^6,17^. This result further strengthens the argument that family history alone should not be used to predict *BRCA* status, or be applied as a criterion to determine eligibility for *BRCA* testing. There is significant variability in the proportion of women with a personal breast cancer history across the different *BRCA*m cohorts in the UK (Table 5), also indicating that personal cancer history alone is also not a reliable predictor of *BRCA* status.

### Impact of *BRCA*m testing on patient management and access to PARP inhibitors

All 34 patients with identified *BRCA* mutations were offered genetic counselling, which was attended by 31 patients. Where appropriate, patients were offered enhanced breast surveillance, ‘To whom it may concern’ information to be passed on to other potentially-affected family members, and entry onto The Royal Marsden’s carrier register.

In line with other literature sources^5,8,18^, this study highlights that knowledge of a patient’s *BRCA*m status had an impact on the management of their ovarian cancer and their ability to access PARP inhibitor treatment. At the time of this study, NHS-funded PARP inhibitor treatment was only accessible to *BRCA*m patients, and no *BRCA*wt patient at Imperial College Healthcare NHS Trust was eligible for PARP inhibitor treatment outside of a clinical trial or Expanded Access Program. It is likely that discrepancies in the proportion of patients able to access PARP inhibitors across the various studies is due to the restrictions on access to NHS-reimbursed PARP inhibitor treatment at various points in time, and variability in relevant PARP inhibitor clinical trial availability at the time of the different studies. It is hypothesised that where clinical trials were open and/or Early Access Programs/Compassionate Use Programs initiated by the sponsoring company were available, a greater percentage of *BRCA*m patients may have been able to access PARP inhibitor treatment allowing for improved patient outcomes across the cohort. When considering the 15 (44.2%) *BRCA*m patients who have not yet relapsed, the knowledge of their *BRCA* status may well inform future treatment planning if they do present with disease recurrence.

Although consideration must be given to the low numbers of patients who accessed PARP inhibitors following a *BRCA*m result obtained through ICHMP, it must also be noted that all seven of the patients who had completed their treatment with a PARP inhibitor had a shorter treatment duration than the median time on treatment reported in the respective PARP inhibitor clinical trials^19,25^ (two patients were still undergoing PARP inhibitor treatment at the time of data collection, following recent initiations). One explanation could be the fact that these are ‘real world data’ and patients were not subjected to a controlled clinical trial protocol with extensive follow up. It is also hypothesised that the fact that only three patients (33.3%) received PARP inhibitors after two lines of platinum therapy, whereas six (66.6%) of patients had received three or more prior lines of chemotherapy before receiving a PARP inhibitor contributed to their shorter treatment duration. The proportion of patients treated with PARP inhibitor therapy following fewer lines of therapy was higher in clinical trials such as SOLO2 (56% receiving olaparib after two lines of therapy and 31% receiving PARP inhibitor after three lines)^26^ and ARIEL3 (63% received rucaparib after only two lines of platinum-based chemotherapy)^20^. This is important given that earlier use of PARP inhibitors is considered to be associated with better outcomes. Furthermore, it is unknown whether patients in this cohort had a higher ECOG performance status than those treated in the PARP inhibitor trials (where the majority of patients had an ECOG performance status of 0), and this may have impacted the duration of their treatment. Only one patient discontinued PARP inhibitor treatment due to unacceptable toxicities, which is in keeping with clinical trials results and speaks to the general tolerability of PARP inhibitors^19,25^.

### Patients aged ≥70 years: access to *BRCA* testing and clinical utility of *BRCA*m result

Previous publications argue that age should not be a discriminating factor preventing access to *BRCA*m testing^14,22^, although it is noted that this does seem to occur in clinical practice^14^. Zhang *et al*.^7^ and Alsop *et al*.^6^ report a *BRCA*m prevalence of 8.4% and 8.3% in patients who were aged ≥60 at the time of diagnosis, respectively. Plaskocinska *et al*.^11^ also recommend that in order to meet the 10% threshold required by NICE, women aged ≥70 should only be tested if they have personal or family history of a *BRCA*-associated cancer. However, the results of this study provide evidence against the imposition of an age-based threshold for *BRCA*m testing. These results show a *BRCA*m prevalence of 11.7% patients aged ≥60 at the time of diagnosis, and a BRCAm prevalence of 11.6% in patients who were diagnosed with ovarian cancer aged 70–79 - indicating that patients aged ≥70 still meet the 10% threshold required under NICE Guidelines^23^. Two *BRCA*m patients who were aged ≥70 at the time of diagnosis subsequently received a PARP inhibitor – with a further two *BRCA*m patients aged ≥70 at the time of diagnosis in remission, but will possibly receive PARP inhibitors at a subsequent relapse. The access to PARP inhibitors by patients aged ≥70 highlights the continued utility of *BRCA*m testing in an older population.

### Evaluation of mainstreaming approach

The ICHMP has successfully tested 255 patients over a period of two years, with 34 *BRCA*m patients identified, providing further proof-of-concept of the oncologist-led mainstreaming model in an NHS centre. In the two years prior to the establishment of the ICHMP only 14% of patients underwent germline *BRCA* testing (these patients were tested via a direct referral to the regional genetics centres, where the mean turnaround time from referral to result was 148.2 calendar days (range 98–175)). However, following the implementation of the ICHMP, 95% of patients had obtained a germline *BRCA* result and this was impactful on patient management. The result of a mean turnaround time (from sample acquisition to receipt of result by treating oncologist) of 20.6 calendar days (range 11–42) highlights one of the key benefits of the IHCMP. The IHCMP allowed a reduction in mean turnaround time from 148.2 days to 20.6 days. This is particularly significant given the outcomes of the SOLO1 trial^21^, and the recent licence extension to allow maintenance treatment with olaparib in the first-line setting. The IHCMP allows the majority of patients to undergo germline *BRCA* testing at their first consultation with their oncologist, and the mean turnaround time of 20.6 days is a sufficient to enable the treating oncologist adequate time to make choices around the most appropriate therapy in this setting. This includes making informed decisions regarding treatment with olaparib where indicated, and alternatively, bevacizumab, in cases where treatment with olaparib is not appropriate. The study also demonstrated the feasibility of a group consenting process for *BRCA* testing that can be used within a ‘mainstreaming’ program and that could be reproduced in other centres with a large pool of untested patients that may be eligible for testing or that may require testing in a timely manner.

### Limitations of this study

As this study was based on patient record data, in some cases not all data points were recorded accurately or available to be included in analysis. In addition, it was evident that not *every* patient under the care of the gynaecological oncology team had been offered *BRCA*m testing through the ICHMP – it should be considered that some patients may have declined testing, or not been offered testing due to clinician bias or error. Although prior to the ICHMP *BRCA* testing was not routine clinical practice at this centre, low numbers of patients under the care of this team had received *BRCA* testing before the implementation of the ICHMP. Some caution should be applied when interpreting this data as it cannot be said with certainty that this is representative of the entire ovarian cancer cohort at this centre within the stated timeframe. Grant funding for somatic *BRCA* testing was not available during this study and NHS funding for such testing is still not available within our oncology clinics in the UK. This study aimed to assess the role of germline *BRCA* testing via a ‘mainstreaming’ process but we acknowledge that somatic *BRCA* testing would have further impact on a small group within this patient cohort in terms of identifying those patients with a somatic *BRCA* mutation who may also have derived benefit from a PARP inhibitor.

### Conclusions & future directions

This study has examined the epidemiology of the *BRCA*m and *BRCA*wt cohorts within the Imperial College Healthcare NHS Trust and concluded that it is highly similar to other cohorts examined across the UK^5,8,11^. It is evident that there is significant impact of the knowledge of the patients’ *BRCA* status on clinical decision-making, as demonstrated by patient access to PARP inhibitors (through NHS-funded olaparib, restricted to *BRCA*m patients - or through Expanded Access Programs for *BRCA*m patients) or patient eligibility to enter clinical trials. Additionally, the knowledge of a patient’s *BRCA*m status has triggered referral to genetic counselling, providing additional management of the *BRCA*m patient as well as an introduction to cascade testing for related family members. The fact that these outcomes were achieved for this cohort provides evidence of the utility and success of an oncologist-led *BRCA* testing mainstreaming service, as implemented at Imperial College Healthcare NHS Trust.

Following the introduction of PARP inhibitors to the clinic, a patient’s *BRCA* status is of greater significance than ever before. Previous publications cite that the time between diagnosis and obtaining a *BRCA*m result has not had an impact on the selection of therapy, as patients tested were generally undergoing primary treatment or in remission^17^ – but with the recent approval of olaparib in the first line treatment setting for *BRCA*m patients and the significant progression-free survival benefits and trends towards a greater overall survival that can be derived from this^21^, clinicians will need to ensure that *BRCA* testing occurs at the very outset of disease management and treatment planning. Results must also be returned in a timely manner so that *BRCA*m patients do not miss the opportunity to benefit from first-line olaparib. The growing body of evidence demonstrating the utility of oncologist-led mainstreaming should be given due consideration by those centres who have not yet achieved routine testing of all non-mucinous ovarian cancer patients, or who are unable to ensure *BRCA* testing is carried out within the recommended timeframes. Furthermore, group education sessions and consenting could be adopted to allow quicker and more efficient testing of untested patients within the ovarian cancer clinics.

As previously mentioned, this study does not look at the testing pathways for somatic (tumour) *BRCA* mutations. In order to optimise clinical management for all ovarian cancer patients, it is key that the testing pathways and processes for germline *BRCA* and somatic *BRCA* mutations are integrated^5^, and both deliver timely results. It is only through up-front, routine testing of all non-mucinous ovarian cancer patients will we be able to ensure that all eligible *BRCA*m patients get the opportunity to benefit from PARP inhibitor therapy.

Finally, future work will assess the impact of the ICHMP on measures taken to reduce risk or prevent other *BRCA*-associated cancers in the *BRCA*m patient cohort, and in the uptake of *BRCA* testing and the consequences thereof in their blood relatives. This is likely to add a further dimension of supportive evidence for the ICHMP and other similar mainstreaming processes.

### Ethics approval and consent to participate

This is a review of a new clinical service within an institution and specific ethical approval was not therefore not sought or required. Patients whose data are described in the study did give informed consent. Patients all signed consent forms that stipulated that they agreed to undertake genetic testing and that the samples, demographics, results and information could be used anonymously for research purposes.

## Data Availability

The datasets used and/or analysed during the current study are available from the corresponding author on reasonable request.
